# The Impact of the Preparation Method on the Properties of Orodispersible Films with Aripiprazole: Electrospinning vs. Casting and 3D Printing Methods

**DOI:** 10.3390/pharmaceutics13081122

**Published:** 2021-07-22

**Authors:** Ewelina Łyszczarz, Witold Brniak, Joanna Szafraniec-Szczęsny, Tomasz M. Majka, Dorota Majda, Marta Zych, Krzysztof Pielichowski, Renata Jachowicz

**Affiliations:** 1 Department of Pharmaceutical Technology and Biopharmaceutics, Faculty of Pharmacy, Jagiellonian University Medical College, Medyczna 9, 30-688 Cracow, Poland; ewelina.lyszczarz@uj.edu.pl (E.Ł.); joanna.szafraniec@uj.edu.pl (J.S.-S.); renata.jachowicz@uj.edu.pl (R.J.); 2 Department of Chemistry and Technology of Polymers, Faculty of Chemical Engineering and Technology, Cracow University of Technology, Warszawska 24, 31-155 Cracow, Poland; tomasz.majka@pk.edu.pl (T.M.M.); kpielich@usk.pk.edu.pl (K.P.); 3 Department of Physical Chemistry and Electrochemistry, Faculty of Chemistry, Jagiellonian University, Gronostajowa 2, 30-387 Cracow, Poland; majda@chemia.uj.edu.pl (D.M.); zych@chemia.uj.edu.pl (M.Z.)

**Keywords:** orodispersible films, electrospinning, 3D printing, solvent casting, aripiprazole

## Abstract

Orodispersible films (ODFs) address the needs of pediatric and geriatric patients and people with swallowing difficulties due to fast disintegration in the mouth. Typically, they are obtained using the solvent casting method, but other techniques such as 3D printing and electrospinning have already been investigated. The decision on the manufacturing method is of crucial importance because it affects film properties. This study aimed to compare electrospun ODFs containing aripiprazole and polyvinyl alcohol with films prepared using casting and 3D printing methods. Characterization of films included DSC and XRD analysis, microscopic analysis, the assessment of mechanical parameters, disintegration, and dissolution tests. Simplified stability studies were performed after one month of storage. All prepared films met acceptance criteria for mechanical properties. Electrospun ODFs disintegrated in 1.0 s, which was much less than in the case of other films. Stability studies have shown the sensitivity of electrospun films to the storage condition resulting in partial recrystallization of ARP. These changes negatively affected the dissolution rate, but mechanical properties and disintegration time remained at a desirable level. The results demonstrated that electrospun fibers are promising solutions that can be used in the future for the treatment of patients with swallowing problems.

## 1. Introduction

Orodispersible films (ODFs) are single- or multilayer stamp-sized polymeric thin sheets, which rapidly disintegrate in the mouth without the need for liquid [1]. They are characterized by a high flexibility of dose adjustment, easy and precise administration, and the ability to adhere to the oral mucosa, which makes them difficult to spit out. These features can be beneficial for pediatric, geriatric, and bedridden patients who have difficulties with swallowing, or psychiatric patients, pretending to take drugs [2,3,4]. The major limitation of ODF is low drug loading capability [2].

During the development of this dosage form, some critical quality attributes should be considered, including dose uniformity, drug release profile, residual water content, disintegration time, and mechanical properties, including thickness, tensile strength, elongation at break, and Young’s modulus [2,3,5]. The disintegration time is indicated as a key characteristic of ODFs affecting their acceptability [6]. Due to the lack of pharmacopoeial standards for the determination of the disintegration time of ODFs, various methods have been introduced. Speer et al. [7] compared different methods and recommended a slide frame and ball method (SFaB), as well as a technique utilizing pharmacopoeial apparatus as the most suitable for the quality control of ODFs. While referring to the mechanical properties, the Ph. Eur. 10. only states that ODFs should “possess suitable mechanical strength to resist handling without being damaged” [1]. Therefore, the methods widely used in the industry for testing the mechanical properties of plastic sheets, i.e., DIN EN ISO 527 [8,9,10] are implemented in the evaluation of ODFs. They define the shape of the specimen and the speed of their extension.

ODFs are composed of water-soluble polymers, such as pullulan [11,12], poly(vinyl alcohol) (PVA) [6,13,14,15,16] cellulose derivates (hydroxypropyl methylcellulose, hydroxypropyl cellulose) [17,18,19,20], or starch [21,22] in which an active pharmaceutical ingredient (API) and other excipients, such as plasticizers, taste-masking agents or surfactants are incorporated [4,23]. They can be manufactured using various methods, i.e., solvent casting, hot melt extrusion, electrospinning, or printing techniques, i.e., inkjet, flexographic, and 3D printing [2,24]. The solvent casting method is the most common technique applied for the preparation of single or multilayer films on a lab-scale as well as industrial continuous production [18,24]. The API is dispersed or dissolved in a polymer solution containing plasticizer or other auxiliary substances and then cast with the defined thickness on an intermediate liner using a film applicator [16,18,20,21,25] or a coating machine [18]. After drying at room or high temperature, the films are rolled up in jumbo rolls, divided into smaller daughter rolls, and finally cut into individual drug units and packed in sachets or other single-dose containers [23].

Electrospinning is a novel technique used in ODFs manufacturing intensively studied in recent years [26,27,28,29,30]. It is a technique based on the electrohydrodynamic process in which an electrified jet of fluid (solution or polymer melt) is stretched and elongated to form continuous fibers. The application of a high voltage leads to the electrostatic repulsion of the surface-like charges and the elongation of the liquid droplet into a Taylor cone as a consequence of the excessive repulsion of similar charges over the surface tension of the fluid. The solvent evaporates quickly, and the fibers form when the jet reaches the collector. The morphology of the fibers depends mostly on the stage of bending instability at which the deposition occurs [31]. The basic requirement that needs to be fulfilled by the macromolecules is their viscosity, which should enable the stretching instead of breaking up into droplets and the ability to carry the electric charge. Various polymers, including PVA, poly(lactic acid) (PLA), poly(ε-caprolactone) (PCL), poly(glycolic acid) (PGA), poly(lactic-*co*-glycolic acid) (PLGA), polyurethane (PU), collagen, pullulan, and chitosan have been successfully electrospun [32].

High permeability, porosity, and specific surface area combined with the ability to functionalize and good mechanical properties make the electrospun fibers attractive material for a wide range of applications, including tissue engineering [33], wound dressing [34], and drug delivery [35]. Moreover, the intimate level of mixing between the API and the polymer, which is difficult to obtain via powder blending, combined with a high evaporation rate, facilitates the formation of physically stable amorphous systems exhibiting improved dissolution performance [36]. The formation of PVP-based orally dissolving nets containing amorphous carvedilol (a non-selective beta-blocker practically insoluble in water) was described by Domokos et al. [28]. The authors reported ca. 10-fold improvement in drug dissolution and proved the applicability of electrospun mats to the formation of orodispersible dosage forms. Another research proved that the formation of pullulan-based electrospun ODFs containing isoniazid (an antibacterial agent used in the treatment of tuberculosis) led to a drug amorphization and complete release within 30 s [37].

In recent years, the 3D printing method was also utilized for the fabrication of orodispersible films. It is an additive manufacturing technique relying on the subsequent deposition of each layer of a material, according to the previously designed 3D model [38]. The final product is formed by extrusion or solidification of liquid, semisolid, or powder material. Extrusion-based technologies, with fused deposition modeling (FDM), are the most common among 3D-printing methods applied in the development of drug delivery systems [39]. The FDM involves the application of heat on the filament, which melts in the printer nozzle, and undergoes solidification on the building platform. This approach needs to be preceded by the hot melt extrusion (HME) of drug-loaded filament. It means that the formulation undergoes multi-step thermal stress. On the one hand, this may induce phase transition and dissolution-enhancing amorphization of API. On the other hand, this may lead to degradation of thermolabile compounds. To avoid the exposure of the drug to a high temperature, thermoplastic polymers, such as PVA, PLA, polyvinylpyrrolidone (PVP), cellulose derivatives, or polyurethanes are added.

Generally, the appropriate flexibility of ODFs is achieved by the addition of a plasticizer, which usually penetrates the polymeric network and reduces the cohesive forces among the chains [40]. The plasticizer content is often high, and the decision on its type needs to be deeply analyzed, considering its effect on the formulation, potential toxicity, and environmental hazards. Cast films are characterized by irregular surface morphology and the presence of the crystalline domains resulting from slow solvent evaporation. On the contrary, fast solvent removal occurring in the electrospinning process prevents crystal formation. Moreover, the porosity of electrospun fibers reduces the cohesive forces between macromolecules and provides more space for structural relaxation, and consequently, improves the mechanical properties and plasticity over cast films [41]. Importantly, no plasticizers are then necessary, or their content is much lower than in the case of cast films. Tyagi et al. described the formation of PVA-based mucoadhesive backing films with diphenhydramine. The results revealed that films obtained by solvent casting were brittle unless a high amount of glycerol was introduced. In contrast, electrospun fibers were flexible even with a very low glycerol content [42]. Similar results have been obtained for PVA-PVP orodispersible films containing rizatriptan benzoate, prepared by either casting method or electrospinning. Moreover, the results indicated faster dissolution rates, shorter film disintegration, and superior bioavailability resulting from the nanometric size of the polymer net. Despite the submicron size of the electrospun membranes, the alignment of the fibers can also tune film flexibility expressed by the tensile strength, Young’s modulus, and elongation at break [26]. Randomly oriented fibers collected on static collectors have lower tensile strength than aligned fibers. On the other hand, aligned films obtained using a rotating drum collector express lower tensile strength than their random counterparts [27]. The increase of the mechanical strength of the electrospun fibers can also be achieved by cross-linking the polymer, as proven by the example of PVA [43,44] or gelatin [45] fibers. The cross-linked polymeric fibers are also characterized by higher water resistance, which in the case of ODF films is an undesirable feature.

This work aimed to develop the formulation of orodispersible films using electrospinning technique and compare their properties to ODFs prepared using film casting and 3D printing methods. Aripiprazole (ARP), which is characterized by poor solubility in water (BCS class II), was used as a model drug. It is a third-generation antipsychotic drug, a partial agonist at D_2_ and 5-HT_1_ receptors and antagonist at 5-HT_2A_ receptors used in the treatment of mental or central nervous system disorders, e.g., schizophrenia, bipolar disorder, depression, Tourette’s syndrome, delusional disorders, or irritability associated with autistic disorders in both children and adults [46,47,48]. Given that those diseases as well as active pharmaceutical ingredients (API) used in their treatment are reported to induce dysphagia and xerostomia, difficulties in swallowing conventional dosage forms, i.e., tablets or capsules, may lead to non-compliance or discontinuation of pharmacotherapy [49]. Therefore, formulation of more acceptable dosage forms with aripiprazole, such as orodispersible tablets (ODTs) or ODFs, are proposed [3,50,51].

Depending on the method of preparation, the films differ in structure, thickness, and appearance. However, despite those differences, they should disintegrate rapidly in the mouth and possess good mechanical properties [1].

We compared the films’ morphology, wettability, water content, mechanical properties, disintegration time uniformity of the content, and the dissolution of aripiprazole. The X-ray diffraction (XRD) and differential scanning calorimetry (DSC) was used to examine the crystalline structure of the aripiprazole in films. Mechanical properties such as elongation at break, tensile strength, and Young’s modulus of all ODFs, were measured using a texture analyzer. Disintegration time was tested using two different methods. Additionally, the wetting characteristic was evaluated by contact angle measurement, while the water content was measured using the Karl Fisher titration method. The uniformity of content and dissolution studies were performed with the validated spectrophotometric assay. Moreover, the properties of the ODFs were evaluated after a one-month storage period (25 °C/60% RH) to estimate their stability.

## 2. Materials and Methods

### 2.1. Materials

Aripiprazole (7-{4-[4-(2,3-dichlorophenyl)-1-piperazinyl]butoxy}-3,4-dihydro-2(1H)-quinolinone) was purchased from HyperChem (Zhejiang, China). Poly(vinyl alcohol) (Poval 4-88), which was used as a film forming polymer and was obtained from Kuraray (Tokyo, Japan). Glycerol (85%) was acquired from Fagron (Cracow, Poland) and glacial acetic acid, Hydra-Point Solvent G and Hydra-Point Titrant 5 mg H_2_O/mL, were purchased from Avantor Performance Materials Poland S.A. (Gliwice, Poland). Sodium Acetate Anhydrous, Hydranal^®^-Water Standard 10.0 were bought from Sigma-Aldrich (Sigma-Aldrich, Saint Louis, MO, USA). Distilled water was used to prepare all aqueous solutions.

### 2.2. Preparation of Electrospinning and Casting Solution

According to the preliminary test results (data not shown), 35% *w*/*w* PVA was selected for the preparation of films by electrospinning and casting method. An aqueous PVA solution was prepared by dissolving the polymer in hot water (90 °C) with continuous stirring at 180 rpm using a Heidolph MR HeiTec magnetic stirrer (Schwabach, Germany). The solution of API (20% *w*/*w*) was prepared by dissolving the appropriate amount of aripiprazole in glacial acetic acid. The ARP solution was added to the polymer solution in different weight ratios, given in Table 1, mixed using a glass rod, and left for 1 h to remove air bubbles.

The casting solution was prepared based on the electrospinning solution Esp ODF 3. However, the addition of a plasticizer was necessary to obtain flexible films. Therefore, the glycerol at 10% concentration (*w*/*w* of the polymer weight) was added to the PVA solution prior to the API solution and stirred at 180 rpm at 90 °C for 1 h.

### 2.3. Preparation of Films by Electrospinning

A 50 mL syringe was filled with the electrospinning solution and mounted in a syringe pump (Ascor AP14, Ascor Med Sp. Z o.o., Warsaw, Poland). An injection needle with an internal diameter of 0.6 mm was attached to the syringe through a silicone tube. The positive electrode of a high voltage power supply (E-Fiber EF020 SKE Research Equipment^®^, Bollate, Italy) was connected to the needle, while the grounding electrode was connected to a rotating metal drum collector (24 × 30 cm) wrapped with aluminum foil. The electrospinning process was carried out at room temperature. The electrical potential applied to the needle was 20 kV or 25 kV, and the distance between the needle and the collector was 10 cm. The feeding rate of the spinning solution was set at 1 mL/h, and the process was conducted for 10 h. Films were dried for 24 h at room temperature and then cut into rectangular strips of 2 × 3 cm, 3 × 3 cm or the shapes corresponding to DIN EN ISO 527-3 (ISO-527-3 2002) standard for mechanical properties evaluation. They were stored for three days at room temperature in a desiccator before testing.

### 2.4. Preparation of Films by Casting Method

The casting solution was poured on the plastic foil mounted on the table of a motorized film applicator (Elcometer 4340, Elcometer, Utrecht, Belgium). The wet film of 500 μm thickness was left to dry at room temperature for 24 h and cut into rectangular strips (2 × 3 cm or 3 × 3 cm), and the shapes corresponding to DIN EN ISO 527-3 [10] for mechanical properties evaluation. All films were stored for 3 days in a climate chamber (HP 105, Memmert, Schwabach, Germany) at 25 °C and 60% relative humidity (RH).

### 2.5. Preparation of Films by 3D Printing

The ODFs with ARP were prepared using the FDM method. Firstly, a filament loaded with ARP was prepared by hot-melt extrusion. Aripiprazole and PVA in a weight ratio of 3.5:96.5 were mixed, moistened with ethanol, and dried up at 60 °C for 6 h. The filament with ARP was prepared using a 40D, 12-mm co-rotating twin-screw extruder (RES-2P/12A Explorer, Zamak Mercator^®^, Skawina, Poland). The powder blend was fed with a gravimetric feeder MCPOWDER^®^ (Movacolor^®^, Sneek, The Netherlands) at approximately 75 g/h and extruded through a 1.75 mm die with a screws’ speed of 50 rpm, which resulted in pressure reaching up to 100 bar. The filament was collected with an air-cooled conveyor belt (Zamak Mercator^®^, Skawina, Poland) without stretching. The screw configuration, including conveying sections and three kneading zones, was described in detail in our previous work [52]. The profile of temperature zones is displayed in Table 2.

In a second step, the Blender^®^ 2.79b software (Blender Foundation, Amsterdam, The Netherlands) was utilized to design the 3D model of orodispersible films. The designed objects were 30 mm long, 0.15 mm heigh, and 20 or 30 mm wide, with the shapes corresponding to DIN EN ISO 527-3 standard [10] for mechanical properties evaluation. The 3D model of ODFs created with the Blender^®^ software was exported to the STL file format, describing the triangular geometry of the 3D object in the form of XYZ coordinates.

Finally, the 3D model of the ODF was exported to the Voxelizer^®^ slicing software (version 1.4.18, ZMorph, Wroclaw, Poland), and films were printed using a patch width of 0.3 mm. The layer height was set at 0.15 mm, which was equal to the high of the films. Two outlines and honeycomb infill with 40% density were designed for film printing. The ODFs with aripiprazole were printed using an FDM ZMorph^®^ 2.0 S personal fabricator (Wroclaw, Poland) equipped with a 1.75 mm single printhead, with a 0.3 mm nozzle. The printing temperature was 190 °C, the temperature of the building platform was 65 °C, and the printing speed was 10 mm/s. After the printing, the films were stored for three days in a Memmert HP 105 climate chamber (Memmert, Schwabach, Germany) at 25 °C and 60% RH before further analyses were performed.

### 2.6. Viscosity of the Electrospinning and Casting Solution

The rheological tests were performed using a R/S Plus Rheometer (Brookfield Ametek, Harlow, UK with a C75-1 rotor, using the plate-to-plate arrangement. The measurements of viscosity were made using the Controlled Rate (CR) program with the rate of 6 s^−1^, during 300 s at a temperature of 25 °C. Ten repetitions were made for every sample, and their values were averaged.

### 2.7. Scanning Electron Microscopy (SEM)

The fibers morphology, during the optimization of the electrospinning process, was investigated by a Phenom Pro (Thermo Fisher Scientific, Grand Island, NE, USA) scanning electron microscope. The accelerating voltage of the beam was equal to 10 kV. Small pieces of the films (ca. 0.5 × 0.5 cm) were placed on the SEM conductive adhesive tape previously glued to a specimen mount. Microphotographs were taken at magnifications of 2000×.

The morphology of the electrospun, cast, and printed films was examined using an S-4700 scanning electron microscope (Hitachi, Tokyo, Japan), due to its higher resolution. The accelerating voltage of the beam was equal to 20 kV. Small pieces of the films (ca. 0.5 × 0.5 cm) were placed on the SEM conductive adhesive tape previously glued to a specimen mount. The electrospun films were sputtered with gold. Microphotographs were taken at magnifications of 2000×, 5000×, and 10,000× in the case of electrospun films, and 50×, 250×, 500×, and 2000× in the case of cast and printed films.

### 2.8. Optical Microscopy

The morphology of cast and 3D printed films was examined using a stereoscopic microscope MST 200M (PZO, Warsaw, Poland) and a polarized light microscope (Hund Wetzlar H600, Hund Wetzlar GmbH, Wetzlar, Germany). Digital images were captured by a TPL MicroCam 5MPx or an OPTA-TECH 16M (OPTA-TECH, Warsaw, Poland) at magnifications of 6×, 37×, and 100×.

### 2.9. Differential Scanning Calorimetry (DSC)

The thermodynamic properties of raw ARP and prepared films were examined using a DSC 1 STAR^e^ System (Mettler-Toledo, Greifensee, Switzerland). Samples were heated in argon atmosphere (80 cm^3^/min) within the range of 25 °C and 180 °C, at a heating rate of 10 °C/min. Measurements were performed in an aluminum open pan. Melting points were determined as the onset of the peak.

### 2.10. X-ray Diffraction (XRD)

The crystalline structure of the samples was analyzed at ambient temperature using a Mini Flex II X-ray diffractometer (Rigaku, Tokyo, Japan). Diffraction patterns were collected over 2θ range between 3° to 43° with 5°/min step. Samples were measured as received.

### 2.11. Film Thickness and Weight

To determine film mass, 10 randomly taken 2 × 3 cm samples were weighed with an MS 105DU analytical balance (Mettler-Toledo, Greifensee, Switzerland). Thickness uniformity was measured using a micrometer screw (Mitutoyo, Kawasaki, Japan).

### 2.12. Contact Angle

The sessile drop technique was applied to determine the wettability of ODFs using a DSA255 drop shape analyzer (Krüss, Hamburg, Germany). The droplet of the distilled water of volume equal to 2 µL was deposited on the surface of the ODFs. All the measurements were carried out in triplicate.

### 2.13. Water Content Using Karl Fisher Titration

The water content in the films was determined using an 870 KF Titrino Plus potentiometric titrator (Metrohm, Herrisau, Switzerland), calibrated with 1.0 g of Hydranal^®^-Water Standard 10.0. The used solvents included a Hydra-Point Solvent G- methanolic imidazole solution and sulfur dioxide and a Hydra-Point Titrant 5 mg H_2_O/mL—iodine methanol. Prior to the test, the film samples weighing ca. 1.0 g were milled with dry ice to avoid their stickiness using an Tube-Mill 100 high-speed mill (IKA, Königswinter, Germany) for 1 min at 15,000 rpm. The samples were accurately weighed (ca. 0.2 g) and put into a titration flask filled with the Hydra-Point Solvent G and titrated with the Hydra-Point Titrant 5 mg H_2_O/mL. All measurements were carried out in triplicate.

### 2.14. Mechanical Properties

Mechanical properties of films were determined using an EZ-SX texture analyzer (Shimadzu, Kyoto, Japan) with a 20 N load cell. Test specimens of type 5 were prepared according to the DIN EN ISO 527 standard [10]. Films were placed between the jaws of the apparatus and extended with a 5 mm/min speed until they broke. Young’s modulus, tensile strength (TS), and elongation at break (%E) were determined with Trapezium X software (Shimadzu, Kyoto, Japan). The test was performed for the five samples of each formulation.

### 2.15. Disintegration Time

The disintegration time of the films was evaluated using a slide frame and ball method and a pharmacopoeial apparatus [7]. In the first method, film samples of 3 × 3 cm were fixed in a holder with a circular hole of 10 mm diameter and stainless-steel ball (d = 10 mm, mass = 3.5 g). Then, 900 μL of distilled water at 37 °C were placed on their surface. The time until the ball penetrated through the film was recorded.

In the second method, film samples of 2 × 3 cm were burdened with magnetic clips (3 g) and mounted into the holder clips attached to the tubes of pharmacopoeial disintegration apparatus type A (ED-2 SAPO, Electrolab, Mumbai, India). The test was performed using 900 mL of distilled water maintained at 37 °C. The disintegration time of the films was recorded when the bottom clips dropped down. Both tests were performed for the six samples of each formulation.

### 2.16. Wetting Assay

A 2 × 1.5 cm sample of ODF prepared by the electrospinning method was placed in a plastic dish with a diameter of 5.5 cm filled with 5 mL of purified water at room temperature. The disintegration and dissolution of the ODF mats were recorded using a camera with 33 frames per second.

### 2.17. Uniformity of Content

Ten randomly taken films of each formulation were accurately weighed and shaken with 25 mL of water at 70 °C for 12 h (WNB 22, Memmert, Schwabach, Germany). After cooling, 50 mL of ethanol was added and shaken for another 4 h at room temperature. After dilution, drug concentration was assayed spectrophotometrically at λ = 250 nm using a UV-1800 spectrophotometer (Shimadzu, Kyoto, Japan).

### 2.18. Dissolution Studies

The dissolution studies were performed in accordance with the FDA guidance for aripiprazole ODTs using a pharmacopoeial apparatus type II (Vision G2 Elite 8, Hanson Research, Chatsworth, CA, USA) equipped with a VisionG2 AutoPlus autosampler (Hanson Research, Chatsworth, CA, USA). The ODF films were inserted into the stainless-steel spring-like sinkers and placed in 1000 mL of acetate buffer of pH = 4.0 ± 0.05 at 37 °C. The paddle rotation speed was 75 rpm. Filtered samples were withdrawn at 5, 10, 15, 30, 45, 60 min and analyzed spectrophotometrically at λ = 250 nm (UV-1800 spectrophotometer, Shimadzu, Kyoto, Japan) using a flow-through 10 mm cuvettes. The tests were carried out in triplicate, and the results represent the average with their standard deviation (SD).

### 2.19. Stability Study

The ODFs were placed in aluminum sachets, sealed and stored under controlled conditions for one month at 25 °C and 60% RH. To determine the stability of the ODFs the physicochemical properties, mass, thickness, mechanical properties, disintegration time, and dissolution behavior were evaluated.

### 2.20. Statistical Analysis

Statistical analysis was performed with IBM^®^ SPSS^®^ Statistics software (version 27.0.1.0, IBM, SPSS Inc., Warsaw, Poland). All data were expressed as mean ± standard deviation (SD). The results were compared by one-way analysis of variance (ANOVA) followed by Tukey’s honestly significant differences (HSD) post hoc test or with Student’s *t*-test when only two means were considered. Statistical significance was accepted for *p* < 0.05.

## 3. Results and Discussion

In our study, the PVA, a commonly used film-forming polymer [16,26,53,54,55], due to its good water solubility and rapid disintegration/dissolution in the mouth after application, was selected as a polymer for the preparation of ODFs, i.e., electrospinning, casting, and 3D printing. Based on the preliminary studies, the PVA solution at a concentration of 35% was chosen for electrospinning. Lower concentration (below 30%) resulted in beaded morphologies, and a higher concentration caused needle clogging. Aripiprazole is a weak alkaline drug with good solubility in acidic solutions [56]. Thus, it was dissolved in glacial acetic acid before the addition to the polymeric solution. Cast films were prepared using a similar solution as for electrospinning, except that glycerol was additionally used at a concentration of 10% (1/10 of the polymer mass) to ensure appropriate flexibility of films after drying. Its addition has no visible effect on the viscosity of the polymer solutions. The value of this parameter measured at a shear rate of 6 s^−1^ was 2.710 ± 0.046 Pa·s in the case of solution used for electrospinning and 2.790 ± 0.037 Pa·s in the case of the cast solution. The 3D printed films, prepared using the FDM method, were also PVA-based. Apart from the polymer, they contained only ARP, without any additional excipients.

### 3.1. Optimization of the Electrospinning Process

**Figure 1:**
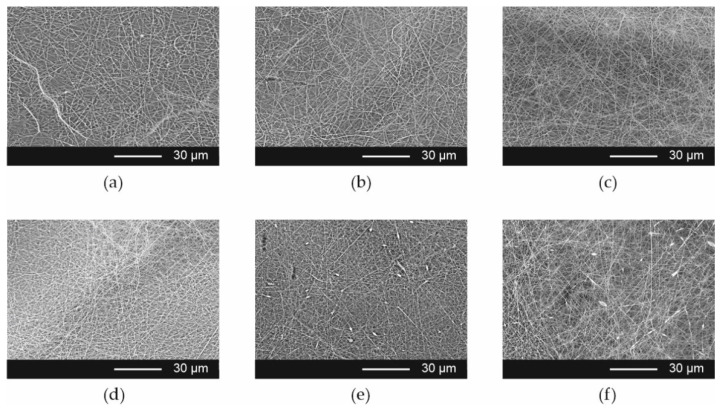
Scanning electron microscopic images of electrospun fibers ((**a**) Esp ODF placebo; (**b**) Esp ODF 1; (**c**) Esp ODF 2; (**d**) Esp ODF 3; (**e**) Esp ODF 4; (**f**) Esp ODF 5; process parameters: 25 kV, 1.0 mL/h, 10 cm; magnification 2000×).

The influence of the composition of the electrospinning solutions and process parameters on the fiber morphology was evaluated. The SEM data has shown that smooth fibers without bead formation were obtained from placebo solution and formulations containing ARP solution in weight ratios 9 + 1, 8 + 2, and 7 + 3, i.e., Esp ODF 1, Esp ODF 2, and Esp ODF 3, respectively. In the case of higher content of the API solution in the electrospinning solutions (Esp ODF 4 and Esp ODF 5), the fibers with bead-on-string morphology were observed, which can be explained by the decrease in polymer concentration in the electrospinning solution (Figure 1). The content of the API in the ODFs and the required drug dose have an impact on the size of the final films, which affects their acceptability [57]. Therefore, to the ODFs preparation by electrospinning process, Esp ODF 3 formulation, further expressed as Esp ODF, was chosen.

The electrospinning process was conducted using two voltage values, i.e., 20 kV and 25 kV. It was found that a lower voltage led to the formation of fibers with beads, while the higher one resulted in smooth fibers.

### 3.2. Morphology of the Films

Based on visual inspection, all the prepared films were homogenous. The electrospun ODFs (Esp ODFs) were white in color, smooth, soft, and fluffy due to their fibrous structure. The surface of cast films (Cast ODFs) was smooth and delicate, while in the case of the 3D printed ones (3D ODFs) it was rough. Their color was beige and ivory, respectively. 

**Figure 2:**
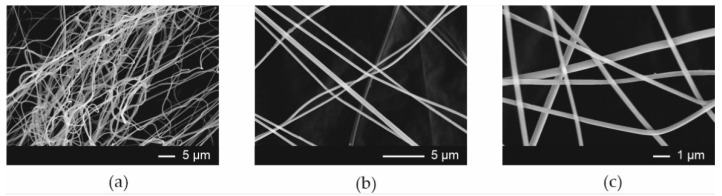
Scanning electron microscopic images of electrospun films (Esp ODF, magnification (**a**) 2000×; (**b**) 5000×; (**c**) 10,000×).

**Figure 3:**
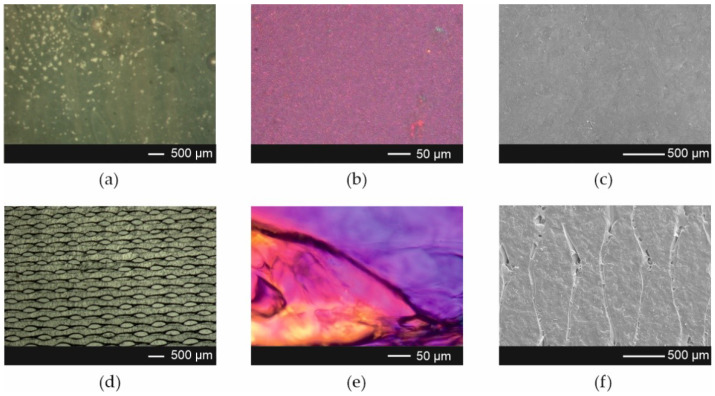
Microphotographs of the Cast ODFs (upper panel) and 3D ODFs (lower panel) (**a**,**d**) visible light image; (**b**,**e**) polarized light image; (**c**,**f**) SEM image at magnification 50×.

The SEM evaluation of the morphology of the Esp ODFs reveals a smooth fiber surface, without bead formation (Figure 2a–c), with a mean diameter of 0.320 ± 0.019 µm. Microphotographs revealed that ARP crystals were present in films prepared by the casting method (Figure 3a–c). The films obtained using 3D printing exhibited a regular porous structure (Figure 3d–f).

The physical parameters of the films are summarized in Table 3. The Esp ODFs had about 10 times lower mass and 3 times lower thickness, as compared with the Cast ODFs and 3D ODFs. Their thickness was also almost 2 times lower than PVP-fibers mats with paracetamol, as reported by Illangakoon et al. [58], although a higher concentration of the polymer solution was used to prepare the films and a larger amount of solution was applied per unit area of the aluminum foil.

The Esp ODFs also had the highest drug content, equal to 21.42%. However, the drug load per film area was the lowest in the case of electrospun films, 0.45 mg/cm^2^, which resulted from much lower mass and thickness. The lowest drug content was determined in the 3D ODFs, and it did not exceed 5%. Their mean mass was significantly higher and their thickness significantly lower than in the case of the Cast ODF (*p* < 0.05).

The storage of the films for one month in the climate chamber at 25 °C and 60% relative humidity has a visible impact on their appearance and physical properties. It was found that the Esp ODFs became more brittle, fragile, and less fluffy, therefore, their thickness decreased to 53.1 ± 10.3 µm, but the difference was not significant (*p* > 0.05). The mass remained unchanged. The Cast ODFs and 3D ODFs were softer and more delicate than shortly after preparation. The mass of Cast ODF increased to 109.7 ± 4.5 mg (not statistically significant, *p* > 0.05), while their thickness up to 156.1 ± 5.7 µm (statistically significant, *p* < 0.05). The mass and thickness of 3D ODFs have also increased, but the difference was not significant (*p* > 0.05). These slight changes might be caused due to water absorption during the storage.

### 3.3. Differential Scanning Calorimetry (DSC)

**Figure 4:**
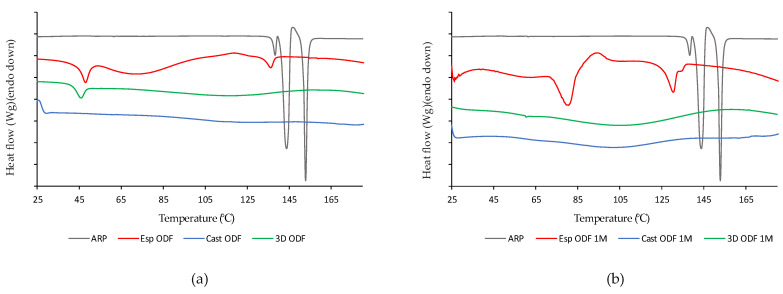
DSC heating curves (**a**) directly after ODFs preparation; (**b**) after one month of storage at 25 °C and 60% RH) of raw ARP (gray line) and ODFs prepared by electrospinning (red line), casting (green line) and 3D printing (blue line) method.

The DSC curves of the ARP and ODFs prepared by electrospinning, 3D printing, and casting methods are presented in Figure 4a. In the case of ARP, the thermogram (gray line) shows three endothermic peaks with onsets at c.a. 137 °C, 141 °C, and 151 °C, which can be associated with melting of forms II, III, and I, respectively [59]. The small exothermic effect, preceding the melting point of form I indicates the phase transition from form III to form I [60].

The analysis of the thermograms of the Cast ODFs (green line in Figure 4a) and 3D ODFs (blue line) indicates full amorphization of the ARP. The endothermic peak with onset at 41 °C, observed for the 3D printed films, originates from phase transition occurring in PVA [61]. In the case of the Esp ODFs (red line), three endothermic events are visible on the thermogram, i.e., an endothermic peak at 40 °C corresponding to the phase transition of PVA, the broad peak at c.a. 60 °C related to the water evaporation, and the peak at 130 °C corresponding to the melting of ARP.

To assess the physicochemical stability of the films, the DSC analysis was also performed after one month of their storage in the climate chamber at 25 °C and 60% RH (Figure 4b). The Cast ODFs did not change during storage. In the case of 3D ODFs, the previously observed peak of PVA phase transition almost disappeared. However, the most visible changes are seen for electrospun ODFs. The DSC curve of the Esp ODF shows the endotherm with an onset at 71 °C reflecting water evaporation and a small exothermic effect at 86 °C, suggesting that the ARP recrystallization and two endothermic events are associated with melting of the ARP.

### 3.4. X-ray Diffraction (XRD)

ARP is one of the most polymorphic drug substances [62]. Such diversity of its forms results from the flexibility around butyl chain connecting aromatic side groups. Braun et al. [63,64] described five polymorphs of aripiprazole, i.e., X°, II, and I, characterized by thermodynamic stability at room temperature, and forms III and IV, which are metastable in the entire temperature range. However, the authors proved that their kinetic stability at room temperature can last for at least a year. They concluded that the metastable forms may then be successfully applied for the development of solid dosage forms containing aripiprazole [60,63]. In 2009, Teva Pharmaceutics issued a US patent disclosing 10 polymorphs of ARP [65]. In 2012, Brittain reviewed 12 pure-phase polymorphs and 8 solvatomorphs [66]. However, one of the described forms, depicted as VI, was further confirmed to be a propylene glycol hemi-solvate by Zeidan et al. [67]. They also discovered a new crystal form, which was called the 12th polymorph of ARP. The reported structure accounts for the total amount of 12 reported non-solvated polymorphs and 9 solved crystal structures of ARP.

**Figure 5:**
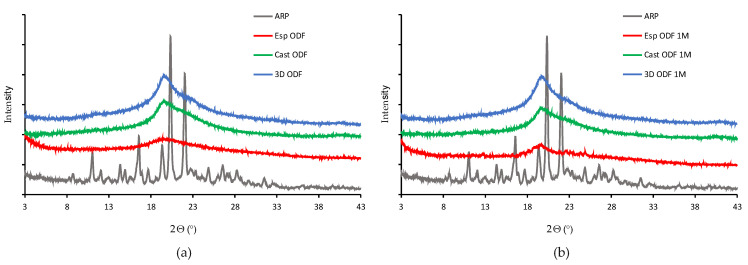
XRD diffraction patterns (**a**) directly after ODFs preparation; (**b**) after one month of storage at 25 °C and 60% RH) of raw ARP (gray line) and ODFs prepared by electrospinning (red line), casting (green line), and 3D printing (blue line) method.

Sharp Bragg peaks registered for raw ARP at 11.04°, 14.36°, 16.58°, 19.34°, 20.34°, 22.04° indicate that the drug exists as a form III polymorph (Figure 5), according to the Cambridge Crystallographic Data Centre (CCDC), deposition number 690585 [68]. It crystallizes in a triclinic crystal system, space group P 1 (2), having the following cell parameters: (a) 10.220(2)Å; (b) 12.208(2)Å; (c) 18.837(4)Å, α 82.28(3)° β 82.52(3)° γ 82.88(3)°.

The lack of Bragg peaks in the diffractograms collected for ODFs containing ARP indicates that the drug undergoes amorphization, regardless of the method of obtaining the film. None of the investigated samples exhibit a distinctive diffractive pattern, which is due to a lack of long-range order of the atoms. Instead, broad amorphous halos were registered (Figure 5a). It is worth noting that in the case of electrospun films, the diffractograms indicate full amorphization of the sample, while thermograms revealed a small melting peak, indicating the presence of crystalline fraction. This difference results from the different sensitivity of both methods and the way how each one analyzes the sample. In DSC, the measured sample is melted, and the information comes from the whole used volume of the material. In XRD, the X-ray beam interacts with only a small spot on the sample surface. If the sample is not homogenous, i.e., if it contains crystalline domains, the beam may not hit them. Also, the sensitivity of XRD is lower than DSC in the determination of crystalline material; depending on the used device, it cannot detect the crystallinity below several percent. Such a limitation does not occur for the DSC. Yet, a small melting peak of aripiprazole is visible in the DSC, and no Braggs peaks indicating the presence of a crystalline phase in the diffractogram is observed.

The XRD analysis performed after one month of film storage demonstrated that the Cast ODFs and 3D ODFs were stable, and ARP was in an amorphous form, which was indicated by broad halos in the diffractogram (Figure 5b). The Esp ODFs were less physically stable. The Bragg peaks were imposed on the amorphous halo. However, due to their low intensity, it was difficult to identify the polymorphic form.

### 3.5. Mechanical Properties of ODFs

Due to the lack of pharmacopoeial guidance for the determination and evaluation of mechanical properties of the ODFs, the test was performed according to standard DIN EN ISO 527 [8,9,10]. The acceptance criteria proposed by Visser et al. [69], i.e., Young’s modulus <550 N/mm^2^, tensile strength (TS) >2 N/mm^2^, and elongation at break (E%) >10% were adopted. All the prepared films met the proposed requirements. However, their mechanical properties differed depending on the method of preparation (Table 4).

The Esp ODFs were the stiffest and least stretchable. They exhibited the highest value of Young’s modulus (242.1 ± 85.5 N/mm^2^) and the lowest value of the percentage of elongation (17.2 ± 1.6%). In the case of the 3D ODFs and Cast ODFs, the values of Young’s modulus were almost 3.5 and 8 times lower, while the values of the percentage of elongation were 2 and 5 times higher, respectively. Much greater elasticity and ductility of the Cast ODFs can be explained by the presence of glycerol and their continuous structure.

The highest maximum force required to break the films was registered for 3D printed films, while the lowest was registered for electrospun films, due to their fibrous openwork structure. However, the differences in the values of the tensile strength for all formulations were not statistically significant (*p* > 0.05) (Table 4).

Although after one month of storage, all the mechanical parameters have changed, they still met Visser’s acceptance criteria (Table 4). The Esp ODF became stiffer, more durable, and less stretchable, which was visible in the increased values of Young’s modulus, F_max_, and tensile strength (not significant, *p* > 0.05) as well as by the significant decrease in the degree of elongation (*p* < 0.05). The Cast ODFs and 3D ODFs had better mechanical properties after the storage period, which was represented by the significant increase of every measured parameter (*p* < 0.05), except for the elongation in the case of the 3D ODFs (*p* > 0.05).

### 3.6. Water Content and Wettability

The residual water content in the ODFs may have a significant impact on their mechanical properties and disintegration time. It was reported that a too high water content may result in the formation of sticky films with extended disintegration time and a higher risk of microbial growth [5,70,71].

The residual water content was analyzed three days after their preparation using Karl-Fisher titration. The water content was the lowest in the Esp ODFs (Table 5), below 3%, and the highest in the Cast ODFs, which may result from the presence of glycerol. A similar observation was reported by Borges et al. [5] and Niese and Quodbach [70], who demonstrated a higher water content in film plasticized with glycerol. In the case of the 3D ODFs, the residual water content was 7.9%. The increased water content may be due to the moisture absorption resulting from the increased surface area and high hydrophilicity of PVA [72].

The nature of the film-forming polymer and hydrophilic/hydrophobic properties of API influence the wettability of ODFs [16,73,74,75]. Here, the chemical composition of films was similar, but the method of the preparation of the films was found to affect the wettability of the ODFs.

The Esp ODFs were characterized by the lowest value of the contact angle, i.e., 24.6°, which suggests high hydrophilicity of their surface. For the other systems, the values of the contact angle exceeded 70° (Table 5), indicating a more hydrophobic nature of the films. However, it was observed that water droplet put on the surface of Esp ODF immediately penetrated the sample, while in the Cast ODF and 3D ODF, it remained on the surface for a few seconds. Given that the contact angle technique is designed for the determination of wettability on smooth, solid surfaces, the effect of water absorption of electrospun films may contribute to the distortion of the measurement, which consequently led to observed lower values of the contact angle than in the case of the Cast ODFs or 3D ODFs.

### 3.7. Disintegration Time and Wetting of the ODFs

According to the Ph. Eur. [1], orodispersible films should be characterized by fast disintegration when placed in the mouth. However, there is a lack of information regarding the methods, which can be applied to the determination of disintegration time. Therefore, two methods recommended by Speer et al. [7], i.e., using a pharmacopoeial apparatus and a slide frame and ball method, were applied to measure the disintegration time of the prepared ODFs. However, the SFaB method was more sensitive to variations in films thickness, which is in line with the findings of Speer et al. [7].

**Figure 6:**
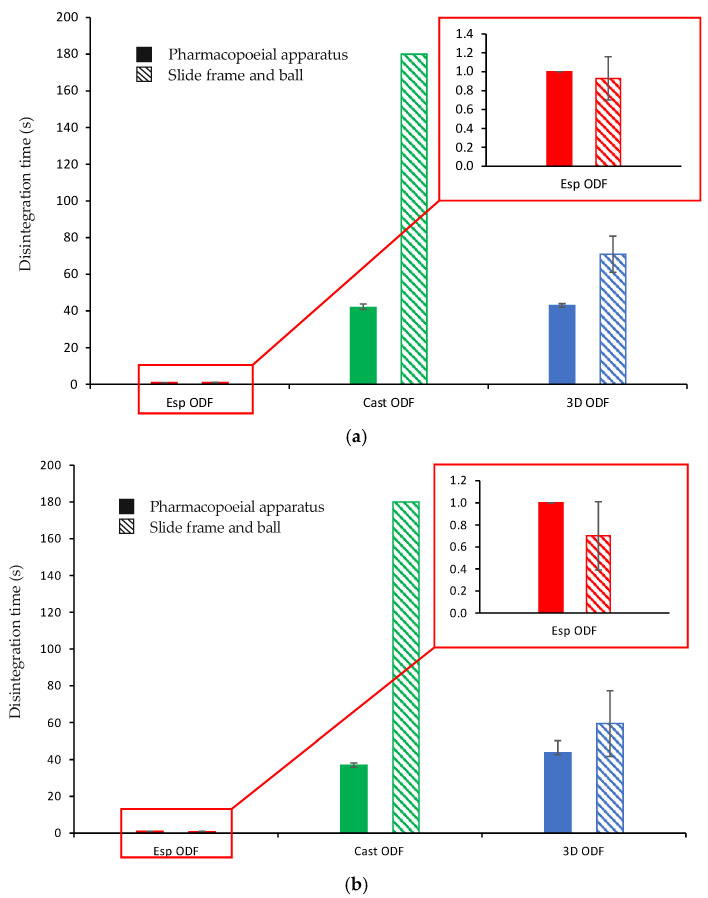
Disintegration time of ODFs (*n* = 6) ((**a**) after preparation; (**b**) after one month storage at 25 °C and 60% RH.

Regardless of the method of disintegration time determination, the electrospun ODFs disintegrated immediately after being in contact with water, i.e., within 1.0 s, as shown in Figure 6a. The disintegration time of the Cast ODFs and 3D ODFs was much longer; however, they were much thicker than the electrospun ODFs. The mean disintegration time for those films, as determined by the pharmacopoeial apparatus, was 41.8 ± 1.9 s and 43.0 ± 1.0 s, respectively. In the case of the SFaB method, the disintegration time for Cast ODF exceeded 3 min and for 3D ODF it was equal to 71.0 ± 9.8 s. Obtained results correspond with those of the contact angle test. The high wettability, highly porous structure, and high surface area of the Esp ODFs influence their rapid disintegration. Similar results, i.e., the faster disintegration time of the Esp ODF than Cast ODF were found by Guo et al. [26] and Tawfik et al. [13].

The disintegration time of all films measured after one month of storage has slightly changed (Figure 6b). The Esp ODFs were still characterized by the fastest disintegration of about 1.0 s, regardless of the applied method of the disintegration time determination. The disintegration time of the Cast ODFs determined using a pharmacopoeial apparatus decreased to 36.8 ± 1.3 s (significant change, *p* < 0.05). However, it still exceeded 3 min in the slide frame and ball method. In the case of the 3D ODFs, their disintegration times measured using the pharmacopoeial apparatus and the SFaB method were 43.7 ± 6.6 s and 59.5 ± 17.8 s, respectively, which shows a not-significant change (*p* > 0.05).

**Figure 7:**
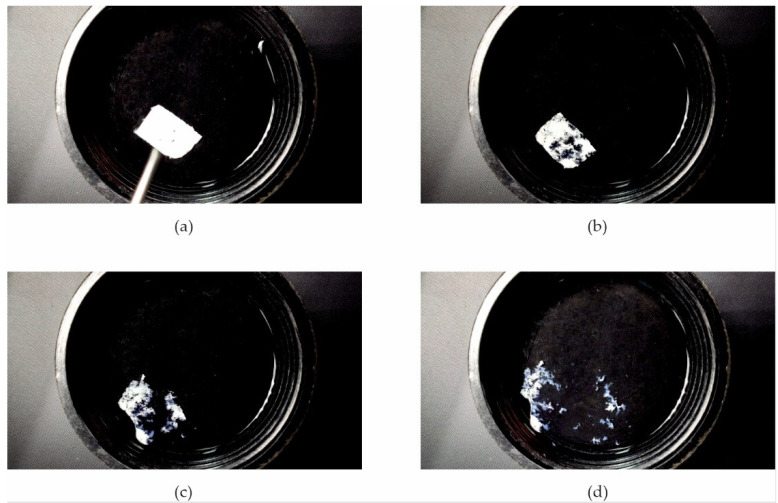
Images of the disintegration of Esp ODF directly after preparation, taken at (**a**) 0 s; (**b**) 1.06 s; (**c**) 1.33 s; (**d**) 1.5 s.

**Figure 8:**
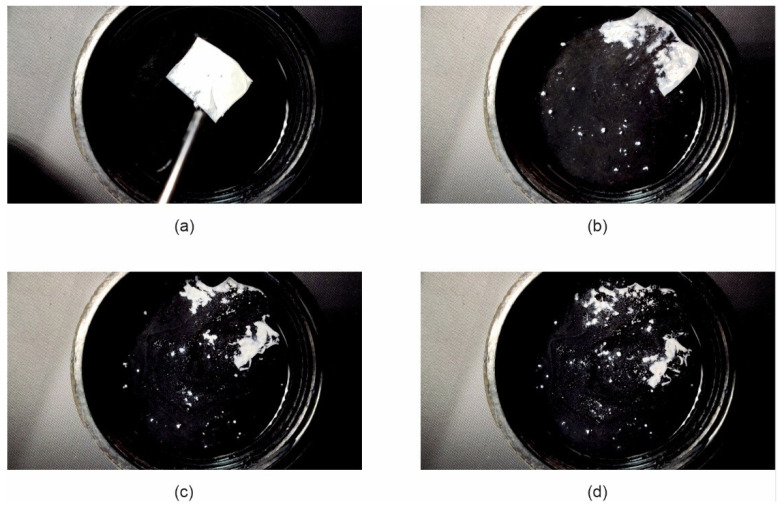
Images of the disintegration of Esp ODF after one month of storage at 25 °C and 60% RH, taken at (**a**) 0 s; (**b**) 1.06 s; (**c**) 4 s; (**d**) 8 s.

Due to the very short disintegration time of the Esp ODF, their wetting behavior when placed in water has been recorded. It was found that immediately after the contact of films with water, they revealed fast hydration and disintegration within 1.5 s (Figure 7). This phenomenon is apparently due to its highly porous structure. Storage of the Esp ODFs extended their hydration time and the elongated disintegration time in this test to 8 s (Figure 8).

### 3.8. Dissolution Study

**Figure 9:**
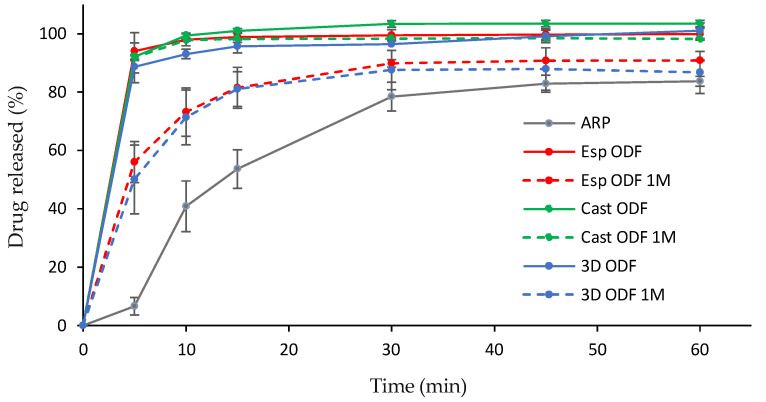
Dissolution profiles of raw ARP (grey line) and ODFs prepared with electrospinning (red line), casting (green line) and 3D printing (blue line) method after preparation (solid line) and after one month storage at 25 °C and 60% RH (dashed lines).

ARP is a poorly water-soluble drug. It is a weak base, which means that its solubility increases with a decrease in pH. According to the FDA Dissolution Database [76], an acetate buffer of pH = 4.0 is suitable for dissolution testing of orally disintegrating tablets containing ARP. Such a medium enables to achieve sink condition in the test and predict the dissolution behavior of the orodispersible dosage form in vivo more accurately than in the case of phosphate buffer having a higher pH [56]. Therefore, acetate buffer was used in the dissolution study. Due to the lack of guidelines on orodispersible films with aripiprazole, we compare the results of the research to orodispersible tablets. According to the pharmacopoeial monography of orodispersible tablets with aripiprazole, 80% of the drug substance should be released up to 30 min [77], nonetheless, FDA recommended sampling times of 10, 20, 30, and 45 min [76]. In our study, we followed these indications. All the prepared films have shown a high dissolution rate and the amount of ARP released from the Esp ODFs, Cast ODFs, and 3D ODFs after 5 min reached 94.0 ± 2.8%, 92.1 ± 0.8%, and 88.7 ± 2.1%, respectively (Figure 9). The dissolution rate from each film was significantly higher than for the aripiprazole alone (*p* < 0.05). It might be the effect of both amorphization of ARP in the films and the presence of the hydrophilic PVA in the formulations, which facilitated wetting of the ARP and its solvation.

The dissolution tests performed after one month of storage revealed about a 2-fold drop down in the amount of dissolved ARP after 5 min in the case of 3D ODFs and Esp ODFs; however, it was still significantly higher than in the case of bulk ARP (*p* < 0.05). After 60 min, the amount of released ARP after film storage was 1.2- and 1.1-fold lower, respectively. The ARP dissolution profile from Cast ODF was not significantly different than directly after the preparation (*p* > 0.05). The variations observed for the electrospun and 3D ODFs may be partially explained by their increased hardness after the storage, and in the case of the Esp ODFs, partial ARP recrystallization.

### 3.9. Effect of Storage on Film Properties, a Summary

Stability studies are an important part of the evaluation of the final dosage form, essential to guarantee their appropriate pharmacodynamic effectiveness and safety. They are also conducted during the development stage to choose the right packaging material, assuring that the properties of the formulation will remain within limits of specification. Here, in order to determine the stability of prepared ODFs, the physicochemical properties, mass, thickness, mechanical properties, disintegration time, and dissolution rate were measured after one-month storage in the constant climate chamber at 25 °C and 60% RH. The results have already been partially described above.

The stored Esp ODFs were stiffer, more brittle, and fragile than directly after the preparation. Their elasticity decreased, while tensile strength and Young’s modulus increased (Table 4). The XRD analysis has shown the amorphization of ARP in the electrospinning process, while the DSC result indicated only partial amorphization, with a noticeable fraction of crystalline form. However, after one month storage, there were signs of recrystallization in both tests (Figure 4b and Figure 5b). Despite these changes, the disintegration time of the Esp ODFs remained very short after one month, and it was still many times lower than for other ODFs (Figure 6b and Figure 8). Although the dissolution rate of ARP decreased, its amount released after 60 min reached 90.8 ± 0.2% (Figure 9).

The Cast ODFs have shown the best stability among prepared formulations. There was no change in either DSC or XRD data (Figure 4b and Figure 5b). Mechanical properties were even slightly better after storage than for freshly prepared samples (Table 4). The major weakness of the Cast ODFs was their long disintegration time, exceeding 3 min in the slide frame and ball method (Figure 6b). However, it did not affect the release of ARP. The dissolution rate was very high shortly after preparation as well as after one-month storage, i.e., more than 90% of ARP was released from the films in 5 min (Figure 9).

The 3D ODFs appeared softer and more delicate after storage, but mechanical analysis has shown an increase in their mechanical properties, such as tensile strength and Young’s modulus (Table 4). The DSC analysis has shown some changes in the glass transition temperature (Figure 4b), but no sign of recrystallization was visible, which is consistent with XRD data (Figure 5b). The disintegration time did not change significantly (*p* > 0.05), but the dissolution rate dropped down similarly as in the case of the Esp ODFs (Figure 6b and Figure 9).

These simplified stability studies allowed us to gather preliminary data on the properties of the prepared films after storage at room temperature. They suggest that the packaging material used for electrospun and 3D printed films needs to maintain the highest possible barrier protection and a high level of stability. The extended stability studies of prepared films stored in different types of packaging materials will be performed in further studies.

## 4. Conclusions

In this study, the PVA-based ODFs with aripiprazole were successfully formulated via electrospinning.

The SEM images demonstrated that the obtained fibers had a smooth surface and average size diameter of 322.6 nm. The properties of the prepared electrospun fibers qualify them as ODF films. In comparison to the Cast ODFs and 3D ODFs, they were characterized by the highest wettability, which is reflected in the shortest disintegration time, resulting from the increased surface area. The short disintegration time of the ODFs is key factor from the patient’s point of view. The Esp ODFs possessed good mechanical properties, nonetheless, the uniformity of the mass and the thickness should be improved. The high surface area of the Esp ODFs and partial drug amorphization had a positive influence on the dissolution rate; more than 94% of ARP were released after 5 min.

The results from the stability studies indicate that the disintegration time of the Esp ODFs remained very short. However, their mechanical properties deteriorated, while in the case of the Cast ODFs and 3D ODFs the mechanical strength increased. In addition, the dissolution rate of ARP in the case of the Esp ODFs decreased, which may result from the drug recrystallization. Further studies will be focused on the electrospinning process in terms of improving the stability of ODFs stored in different types of packaging materials.

Overall, the performed study has shown that the application of electrospinning can bring multiple advantages for the ODF formulations, such as good mechanical properties, and rapid disintegration time, which are indicated as key features affecting the effectiveness of the formulation, thus improving patients’ compliance and acceptability of a drug. Due to the high flexibility of a dose adjustment and rapid disintegration time, ODFs can be used in the individualization of therapy, especially important in mental disorders in children.

## Figures and Tables

**Table 1:** Composition of electrospinning solutions.

Formulation	Content of Electrospinning Solution Components (*w*/*w*)	
	35% PVA	20% ARP
Esp ODF placebo	10.0	-
Esp ODF 1	9.0	1.0
Esp ODF 2	8.0	2.0
Esp ODF 3	7.0	3.0
Esp ODF 4	6.0	4.0
Esp ODF 5	5.0	5.0

**Table 2:** The profile of temperature zones of the RES-2P/12A Explorer twin-screw extruder.

Zone	Temperature (℃)
1	100
2	175
3	185
4	185
5	185
6	170
Die	155

**Table 3:** Physical properties and uniformity of content of aripiprazole in ODF formulations (*n* = 10); film size 6 cm^2^.

Formulation	Mass (mg)	Thickness (µm)	Drug Content	
			(mg/cm^2^)	(% m/m)
Esp ODF	9.4 ± 1.3	57.7 ± 12.6	0.45 ± 0.03	21.42 ± 0.48%
Cast ODF	108.3 ± 4.7	147.7 ± 6.4	2.98 ± 0.12	16.31 ± 0.27%
3D ODF	95.4 ± 12.3	170.1 ± 29.0	0.69 ± 0.09	4.29 ± 0.39%

**Table 4:** Mechanical properties of ODFs (*n* = 5).

Formulation	Young’s Modulus (N/mm^2^)	F_max_ (N)	TS (N/mm^2^)	ε% (%)
**After Preparation**				
Esp ODF	242.1 ± 85.5	3.0 ± 0.4	6.1 ± 0.9	17.2 ± 1.6
Cast ODF	30.3 ± 2.4	5.4 ± 1.2	6.1 ± 1.3	89.6 ± 16.8
3D ODF	69.7 ± 15.0	6.7 ± 1.1	6.0 ± 1.0	30.3 ± 5.7
**1 Month**				
Esp ODF	305.6 ± 102.7	2.5 ± 0.9	7.4 ± 2.7	10.0 ± 2.2
Cast ODF	47.8 ± 8.2	7.7 ± 1.2	8.1 ± 1.2	140.6 ± 22.3
3D ODF	355.84 ± 45.8	14.1 ± 1.2	12.5 ± 1.0	31.8 ± 4.2

**Table 5:** Water content and wettability of the ODFs (*n* = 3).

Formulation	Water Content (%)	Contact Angle (°)
Esp ODF	2.8 ± 0.4	24.6 ± 6.3
Cast ODF	10.9 ± 1.9	76.6 ± 2.4
3D ODF	7.9 ± 1.1	70.3 ± 2.9

## Data Availability

Not applicable.
